# Metagenome Analysis Identifies Microbial Shifts upon Deoxynivalenol Exposure and Post-Exposure Recovery in the Mouse Gut

**DOI:** 10.3390/toxins15040243

**Published:** 2023-03-25

**Authors:** Jing Jin, Chen Zhang, Xiaoxu Ren, Bowen Tai, Fuguo Xing

**Affiliations:** 1 Institute of Food Science and Technology, Chinese Academy of Agricultural Sciences/Key Laboratory of Agro-Products Quality and Safety Control in Storage and Transport Process, Ministry of Agriculture and Rural Affairs of China, 2 Yuanmingyuan West Road, Haidian District, Beijing 100193, China; 2 Division of Toxicology, Wageningen University and Research, Stippeneng 4, 6708 WE Wageningen, The Netherlands; 3 Laboratory of Microbiology, Wageningen University and Research, Stippeneng 4, 6708 WE Wageningen, The Netherlands

**Keywords:** deoxynivalenol, gut microbiota, metagenomic analysis, spontaneous recovery, inulin supplementation

## Abstract

Deoxynivalenol (DON) is one of the most prevalent food-associated mycotoxins, and is known to cause a variety of adverse health effects on human and animals. Upon oral exposure, the intestine is the main target organ of DON. The current study unraveled that DON exposure (2 mg/kg bw/day or 5 mg/kg bw/day) can significantly reshape the gut microbiota in a mouse model. The study characterized the specific gut microbial strains and genes changed after DON exposure and also investigated the recovery of the microbiota upon either 2 weeks daily prebiotic inulin administration or 2 weeks recovery without intervention after termination of DON exposure (spontaneous recovery). The results obtained reveal that DON exposure causes a shift in gut microorganisms, increasing the relative abundance of *Akkermansia muciniphila, Bacteroides vulgatus, Hungatella hathewayi,* and *Lachnospiraceae bacterium 28-4*, while the relative abundance of *Mucispirillum schaedleri*, *Pseudoflavonifractor* sp. *An85*, *Faecalibacterium prausnitzii*, *Firmicutes bacterium ASF500*, *Flavonifractor plautii*, *Oscillibacter* sp. *1-3*, and *uncultured Flavonifractor* sp. decreased. Notably, DON exposure enhanced the prevalence of *A. muciniphila,* a species considered as a potential prebiotic in previous studies. Most of the gut microbiome altered by DON in the low- and high-dose exposure groups recovered after 2 weeks of spontaneous recovery. Inulin administration appeared to promote the recovery of the gut microbiome and functional genes after low-dose DON exposure, but not after high-dose exposure, at which changes were exacerbated by inulin-supplemented recovery. The results obtained help to better understand the effect of DON on the gut microbiome, and the gut microbiota’s recovery upon termination of DON exposure.

## 1. Introduction

*Fusarium* fungi are world-wide producers of a range of mycotoxins. Deoxynivalenol (DON) belonging to the group B trichothecenes, is one of the most prevalent food-associated mycotoxins mainly produced by *Fusarium graminearum* and *Fusarium culmorum*, and frequently contaminates cereals and cereal products [1,2]. Almost half of a total of 26,613 cereal samples collected from 21 European countries were found to be contaminated by DON, with the highest levels observed in wheat, maize, and oat grains [2]. A survey conducted by Mishra et al. [3] summarized the occurrence of DON in food and related human risk assessments in the past 10 years. Their report shows that current DON exposure levels may pose a health risk to consumers, especially children. The concentration of urinary DON in children was higher than that in adults and the elderly, and researchers suggested that necessary measures must be taken to ensure health [1,4]. The most common route of exposure to DON is through dietary intake [3]. DON is known to cause a variety of adverse effects, of which many are related to the gastrointestinal tract and its functioning. The intestinal tract serves as the main target organ [5,6]. The acute effects are, among others, nausea, vomiting, gastro-intestinal (GI) upset, dizziness, diarrhea, and headache [7,8]. Previous studies reported that chronic exposure to DON can cause intestinal toxicity, including induction of intestinal lesions, affecting cell proliferation and differentiation, altering the intestinal barrier function, and it also can cause immunotoxicity, hematotoxicity, and myelotoxicity [7,9,10,11,12,13].

Trillions of microbes with more than 700–1000 different bacterial species reside in the gut, representing the gut microbiota [14]. Many factors such as pH variation, diet, mucus, host immunity, and environmental factors have been shown to influence the biogeography and composition (both abundance and diversity) of bacteria along the GI tract [15]. Xenobiotics are important environmental factors which can interact with gut microbiota [16]. Previous studies reported different effects of DON on the microbiota in different animal models and at different dose levels (0, 2.5, 5, and 10 mg per kg diet) [17]. DON was reported to cause a decrease in total fecal bacterial numbers [11], and changes in the richness (Chao1) and evenness index (Shannon) [11,12]. Notably, DON treatment altered intestinal microbiota, resulting in increases and decreases at the phylum, family, or genus level [13,17]. At the phylum level, the relative abundance of Firmicutes and Proteobacteria in chicken cecal digesta were reported to be increased upon DON exposure [12]. At the family level, in human fecal microbiota-inoculated germ-free rats, the relative abundance of the *Clostridiaceae* family increased, *Ruminococcaceae* remained unaffected, while *Enterobacteriaceae* decreased upon exposure of the rats to DON. At the genus level, the abundance of the *Bacteroides*/*Prevotella* group increased, while the *Escherichia coli* group decreased after feeding rats with DON [13]. In another study, the fecal microbiota of Wistar rats showed the genus *Coprococcus* to be increased upon exposure of the rats to DON [17].

Despite above-mentioned evidence on the effect of DON on gut microbiome composition, our understanding of the effects of DON exposure on gut microbiome function and of the post-exposure recovery process is limited. In this study, we employed a metagenome-wide association approach to identify microbial species and functional shifts that follow DON exposure, and to characterize natural recovery of the microbiome after termination of toxin exposure in the mouse gut microbiota. For the recovery, an intervention with the prebiotic inulin was also included in the studies. Interventions with inulin have been reported to modulate gut microbiota composition and function to promote gut health [18]. However, the impacts of inulin on post-DON exposure recovery of the gut microbiome have not been defined. Therefore, in this study, inulin was added as a dietary supplement to the mouse diet during the recovery period after DON exposure, in order to investigate whether this dietary intervention would facilitate the recovery of the DON-induced disturbances in the mouse gut microbiota as compared to a recovery phase without an extra intervention. The results obtained help to better understand the effect of DON on the gut microbiome, and the gut microbiota’s recovery upon termination of the exposure. The study also aims to understand the role of inulin supplementation in the recovery of the gut microbiome after DON exposure.

## 2. Results

### 2.1. Effects of DON Exposure on Mice Body Weight

**Figure 1:**
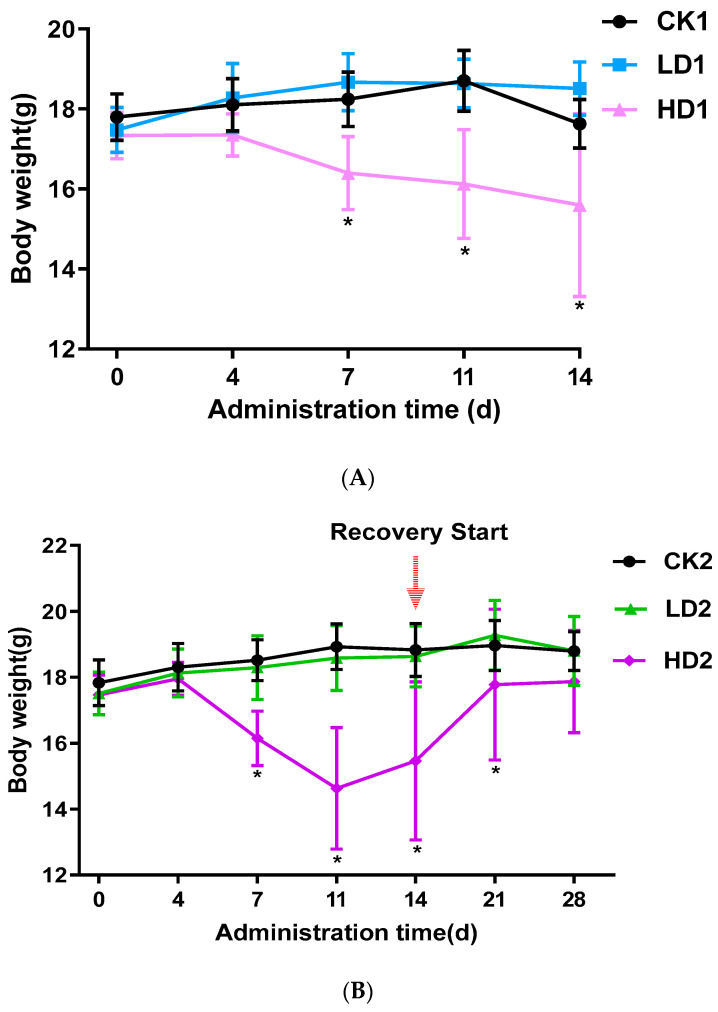

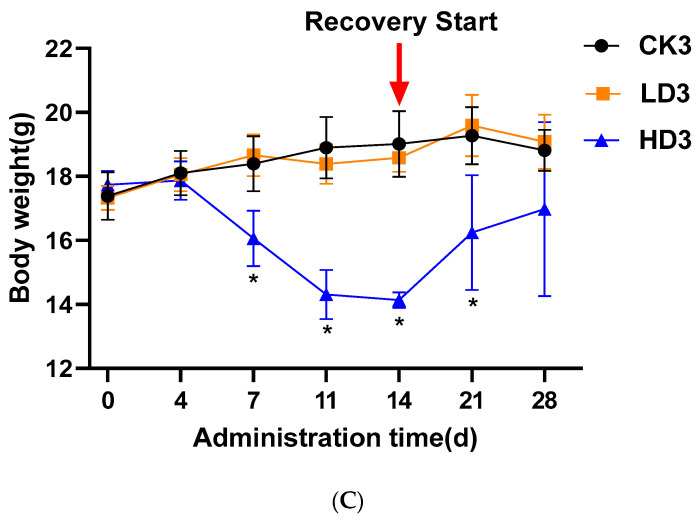
Mice body weight changes during the 2-week DON exposure without (**A**) or with 2 weeks spontaneous (**B**) or inulin-supplemented (**C**) recovery following DON treatment. Data are presented as the mean ± SEM. Mean values were significantly different between the groups (* *p* < 0.05). CKn, LDn, and HDn indicate the control, low-dose, and high-dose DON groups of the respective series.

In the control CON groups (CK1, CK2, and CK3) and the Low-DON groups (LD1, LD2, and LD3), the activity and appetite of mice were normal, with glossy fur, during the 2 weeks with or without low-dose DON exposure. In the High-DON groups (HD1, HD2, and HD3), the body weight of mice was significantly lower. In the second week of administration, High-DON group (HD1, HD2, and HD3) mice were slow in reacting, had dull fur, and had severe diarrhea symptoms. Figure 1A–C shows the changes in mice body weight from the beginning to the end of treatment. A 2-week low-dose (2 mg/kg bw/day) DON exposure did not affect mice body weight, while high-dose (5 mg/kg bw/day) exposure significantly (*p* < 0.05) decreased mice body weight. Both spontaneous and inulin-supplemented recovery groups (HD2 and HD3) gained body weight gradually after high-dose DON withdrawal, but after the 2 weeks recovery, the body weight of mice in these two groups (HD2 and HD3) was still lower than that of the mice in the corresponding control groups (CK2 and CK3) albeit not significantly. In the High-DON groups, the increases in mice body weight during the two weeks recovery showed no difference between recovery in the absence or presence of the prebiotic inulin in the diet.

### 2.2. Microbial Genome Sequencing, Gene Catalog Prediction, and Taxonomic Annotation

**Figure 2:**
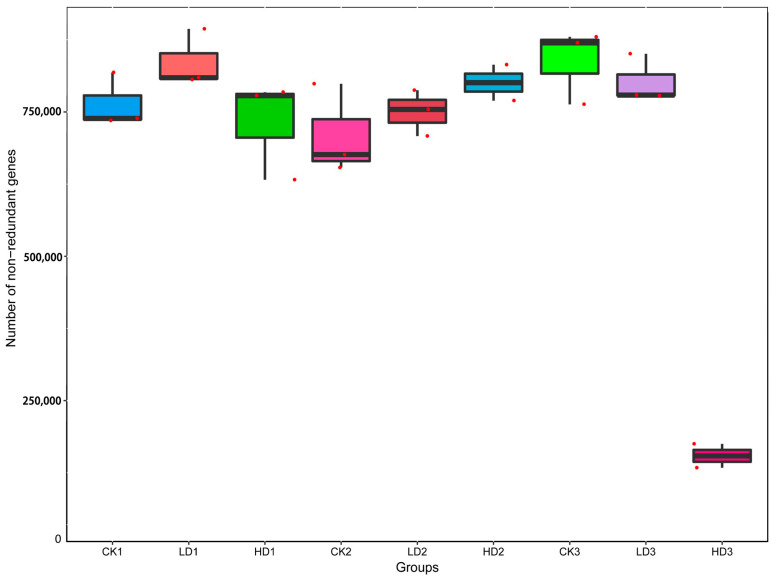
Number of genes identified in the different treatment groups. CKn, LDn, and HDn indicate the control, low-dose, and high-dose DON groups of the respective series. Data are presented as the median and interquartile range.

To explore the effect of DON on intestinal microbiota, the cecum microbiota of the mice was analyzed by sequencing the cecum bacterial metagenome. Supplementary Table S1 presents the amount of raw, clean, and processed data, with the processed data obtained by removing low-quality reads, N reads, and adaptor sequences. Metagenomics Illumina PE150 sequencing yielded 1.35 million genes, and created a set of 1,356,874 Open Reading Frames (ORFs) (Supplementary Table S2), and 52.02% of these were complete ORFs, while 705,888 complete genes were obtained. The assembled sequencing data resulted in a total length of genes at a size of 959.49 Mbp, and an average gene length of 707.13 bp. Of the complete genes, 87.0% were annotated as originating from bacteria, 0.1% from viruses, 0.03% from archaea, 0.2% as unclassified, and 13.0% as unknown. The number of genes varied among different treatment groups based on the assembly genes. Compared with the CON group (CK1), gene numbers of the Low-DON group (LD1) increased, while those in the High-DON group (HD1) decreased, albeit not to a statistically significant extent for both comparisons. Gene numbers of the Low-DON + Recover group (LD2) and the High-DON+Recover group (HD2) increased compared to the CON+Recover group (CK2)—again, not in a statistically significant way. Actually, the gene numbers among groups (CK1) to (LD3) did not show significant differences. Only the gene numbers in the High-DON+Inulin group (HD3) were significantly (*p* < 0.01) reduced compared with each of the other eight groups (Figure 2). This effect was not observed in the CON+Inulin or Low-DON+Inulin group (CK3 or LD3, respectively).

### 2.3. Oral DON Gavage Alters the Composition of Mice Cecum Microbiome

**Figure 3:**
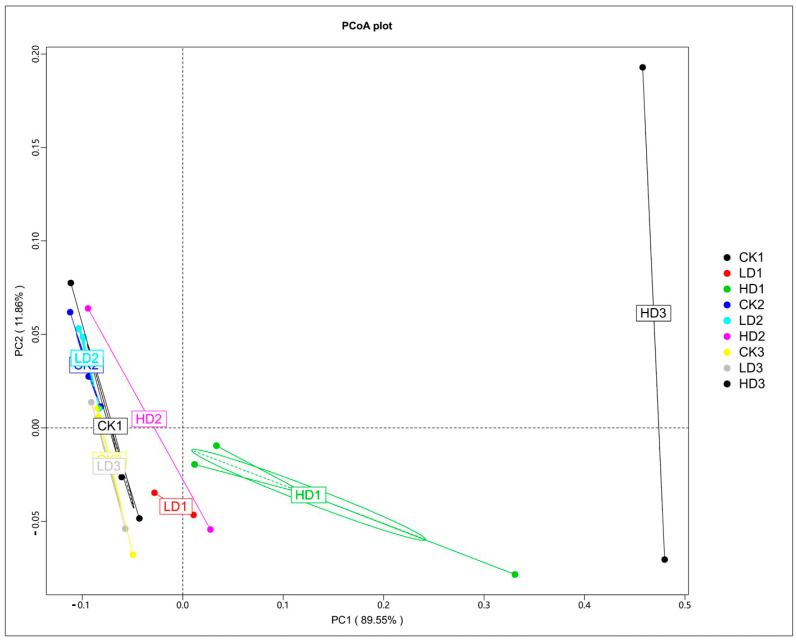
PCoA of Bray–Curtis tax annotation at the phylum level based on species abundance. CKn, LDn, and HDn indicate the control, low-dose, and high-dose DON groups of the respective series.

The effects of DON on the microbiota were studied first by comparing the microbial cecum composition of the low-dose and high-dose DON groups to that of the corresponding controls. To determine the perturbation of the gut microbiota and functional genes by DON exposure, a principal coordinates analysis (PCoA) was performed. The PCoA was based on Bray–Curtis distance, and the principal coordinate combination with the largest contribution rate was displayed. The closer the samples cluster together, the more similar the species composition structure. So, the samples with high community structure similarity tend to cluster together, and the samples with large community differences are far apart. Based on the species abundance of different classification levels, the Bray–Curtis distance matrix was obtained. The PCoA analysis shows that the three control groups, especially CK1 and CK2, clustered very close to each other, which indicated that the mice microbiome is fundamentally stable, and such stability lays the cornerstone of the significance of microbiome composition changes induced by the treatments examined in this study. The PCoA analysis shows a clear separation of the mice cecum microbiota in the controls and low-dose exposure groups, which cluster together, and the three high-dose exposure groups (Figure 3). HD1, HD2, and HD3 deviate from the other clusters, with HD2 separating to a lesser extent than HD1 and HD3. The results demonstrate that DON treatment, especially at high doses, affected the cecum microbial community composition. The results also reveal that following recovery, the DON-treated groups, except for HD3, cluster closer to the respective controls, pointing at adequate recovery. The distance of the HD2 cluster from the control clusters was shorter than that of HD1, suggesting some recovery, while the distance of the HD3 cluster from the control clusters was much greater than that of HD1, suggesting that microbiome composition changes were exacerbated in the HD3 group. The separation between HD2 and HD1 appears less pronounced than that between HD3 and HD1, indicating that in contrast to spontaneous recovery, inulin-supplemented recovery seems to exacerbate the differences.

**Figure 4:**
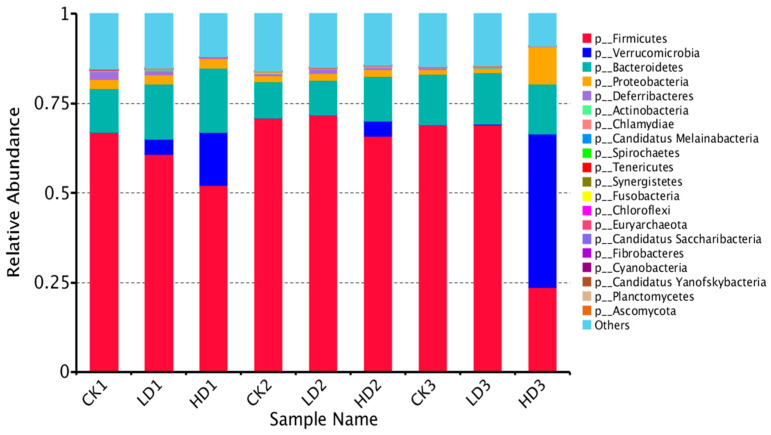
Relative abundance of mice gut bacteria at the phylum level in all treatment groups. CKn, LDn, and HDn indicate the control, low-dose, and high-dose DON groups of the respective series.

A shift in the mouse gut microbiota was observed, and Figure 4 and Supplementary Figure S1A–E show the top 20 identified gut bacteria assigned at the phylum, class, order, family, genus, and species levels from metagenome sequencing reads, with each color representing an individual bacterial species. In terms of the assignment at the phylum level, Firmicutes and Bacteroidetes were the most abundant gut bacteria in all mice groups except the HD3 group, showing notable stability of mice microbiome composition, followed by Proteobacteria, Deferribacteres, and Actinobacteria. Firmicutes were the most abundant in all CON groups (CK1, CK2, and CK3) and Low-DON groups (LD1, LD2, and LD3), while in the High-DON + Inulin group (HD3), Verrucomicrobia was the dominant phylum. Verrucomicrobia seems to be the principal bacteria induced by DON treatment. At the phylum level, Verrucomicrobia and Candidatus Melainabacteria abundance significantly increased with DON dose level. Proteobacteria abundance also increased, but not significantly. The abundance of several phyla, such as Bacteroidetes, Spirochaetes, Tenericutes, Chloroflexia, Euryarchaeota, Chytridiomycota, Nitrospirae, Acidobacteria, Elusimicrobia, and Chlorobi, significantly increased in the Low-DON + Recover group (LD2), but not in the High-DON+Recover group (HD2). At the phylum level, 24 phyla showed significant differences in relative abundance between the CK1 vs. the LD1 group, whereas 32 phyla showed significant differences in relative abundance between the CK1 vs. the HD1 group. Among these species, 13 phyla showed significant differences both in the LD1 and HD1 groups vs. the CK1 group (Supplementary Table S3).

At the genus level, the relative abundance of *Oscillibacter*, *Mucispirillum*, and *Alistipes* decreased, while *Akkermansia*, *Parabacteroides*, *Robinsoniella*, and *Hungatella* increased upon low- and high-dose DON exposure (Supplementary Figure S1D). At the species level, the top relative abundance species were *A. muciniphila*, *H. hathewayi*, *L. bacterium* 10-1, *L. bacterium* 28-4, *F. bacterium* ASF500, *Oscillibacter* sp. 1-3, *M. schaedleri*, *B. caecimuris*, *E. plexicaudatum*, and *L. bacterium* A4 (Supplementary Figure S1E). At the species level, 841 species showed significant differences in relative abundance between the CK1 vs. the LD1 group, whereas 969 species showed significant differences in relative abundance between the CK1 vs. the HD1 group. Among these species, 245 species showed significant changes vs. the CK1 group both in the LD1 and HD1 groups (Supplementary Table S4). Of the top 20 relative abundance species, only *A. muciniphila* showed significantly differences between the LD1 and HD1 vs. the CK1 group.

### 2.4. Spontaneous Recovery Could Mostly Restore the Composition of the Mice Gut Microbiome Changed by DON, but Inulin-Supplemented Recovery Could Not

In order to investigate whether the relative abundance of microbial taxonomic and functional changes after DON exposure were reversible over time, we conducted a study in which the 2 weeks of DON exposure was followed by a 2-week recovery study in which the animals received a standard diet or an inulin-supplemented diet. By day 28, after 2 weeks of spontaneous recovery, at the phylum level, 2 of the 24 above-mentioned identified significant differences between the LD1 and CK1 groups at the phylum level were still significantly different for the LD2 vs. the CK2 group, while 4 of the 32 phyla that were significantly different at the phylum level between HD1 and CK1 were significantly different between the HD2 vs. the CK2 group (Supplementary Table S5). Compared with the CON + Recover group (CK2), the phylum Cyanobacteria remained significantly decreased in the Low-DON + Recover group (LD2) and the High-DON + Recover group (HD2). As described in the above section, at the species level, 841 species showed significant differences between the LD1 and CK1 groups, whereas 969 species were significantly different between the HD1 and CK1 groups. After 2 weeks of recovery, 138 of these 841 identified species were still significantly different between the LD2 and CK2 groups, and 117 of these 969 identified species still showed a significant difference between the HD2 and CK2 groups after 2 weeks of spontaneous recovery (Supplementary Table S6). This indicates that most of the changed gut microbiome species recovered after the 2-week spontaneous recovery. Apparently, these species revive when conditions are once again appropriate. The specific microbial taxonomic profiles were comparable between the CON+Recover group (CK2) and the Low-DON+Recover group (LD2). However, over 10 percent of the DON-altered gut microbiome species in both the LD2 and HD2 groups would not recover after the 2-week spontaneous recovery.

**Figure 5:**
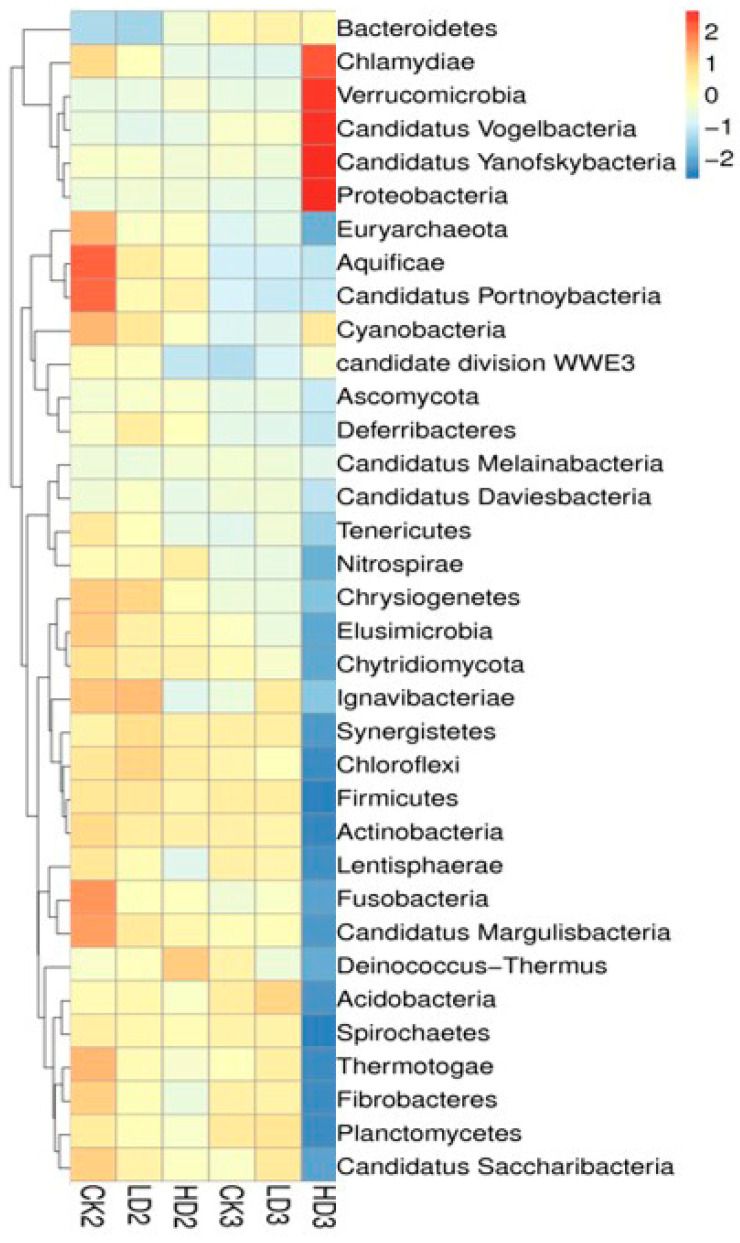
A heat map showing the microbiome composition changes at the phylum level. CK2, LD2, HD2, and CK3, LD3, and HD3 indicate the control, low-dose, and high-dose DON groups with either spontaneous or inulin-supplemented recovery.

In addition to the groups with spontaneous recovery, recovery with dietary inulin supplementation after 2 weeks of DON exposure was also included in the studies in order to assess the effects of dietary supplementation on the regrowth performance of mice gut microbiota. By the end of the 2 weeks recovery under dietary inulin supplementation, the relative abundance between the CON + Inulin group (CK3) and the Low-DON + Inulin group (LD3) did not exhibit significant differences, and none of the 24 above-mentioned identified significant differences at the phylum level still existed. In contrast, 12 of the 32 identified significant differences at the phylum level were still statistically significantly different between the CK3 vs. the HD3 group (Supplementary Table S7). The results suggest that most of the changed microbiome in the mice gut had regrown after low-dose DON exposure under 2 weeks recovery with inulin supplementation, whereas the abundance of the gut microbiome in the High-DON + Inulin group (HD3) was still substantially changed after inulin supplementation compared to the CON + Inulin group (CK3). At the phylum level, the relative abundance of Verrucomicrobia and Proteobacteria increased, and Firmicutes was decreased in the High-DON + Inulin group (HD3) compared to the CON + Inulin group (CK3), albeit no longer to a significant extent. At the species level, 54 of the above-mentioned 841 identified significantly different species between the LD1 and the CK1 groups were still significantly different between the LD3 and CK3 groups after 2 weeks of recovery with inulin supplementation, while after 2 weeks recovery of, in the presence of inulin, 651 of the above-mentioned 969 significant differences remained at the species level between the HD3 and CK3 groups. A total of 10 of these identified significantly different species were still significantly increased in both the LD3 and HD3 groups compared to the CK3 group after 2 weeks of recovery with dietary inulin supplementation (Supplementary Table S8). The relative abundance of the gut microbiome in the CK3 and LD3 groups were similar. A heat map shows the changes in the microbiome composition after spontaneous recovery or inulin supplementation recovery at the phylum level (Figure 5). The results revealed that the changes in the gut microbiome after low-dose DON exposure in mice tended to recover after 2 weeks of recovery both without and with dietary inulin supplementation. However, in the high-dose exposure group, the results turned out to be the opposite. The results indicated that the dominant microorganisms of the mice gut undergoing changes upon high-dose DON exposure do not recover under inulin supplementation to a level comparable to what is observed upon spontaneous recovery. To some extent, inulin supplementation even seemed to result in the opposite effect, counteracting recovery during the recovery period of the microbiome compared to all other groups.

### 2.5. Oral DON Gavage Alters the Function of Mice Cecum Microbiome

**Figure 6:**
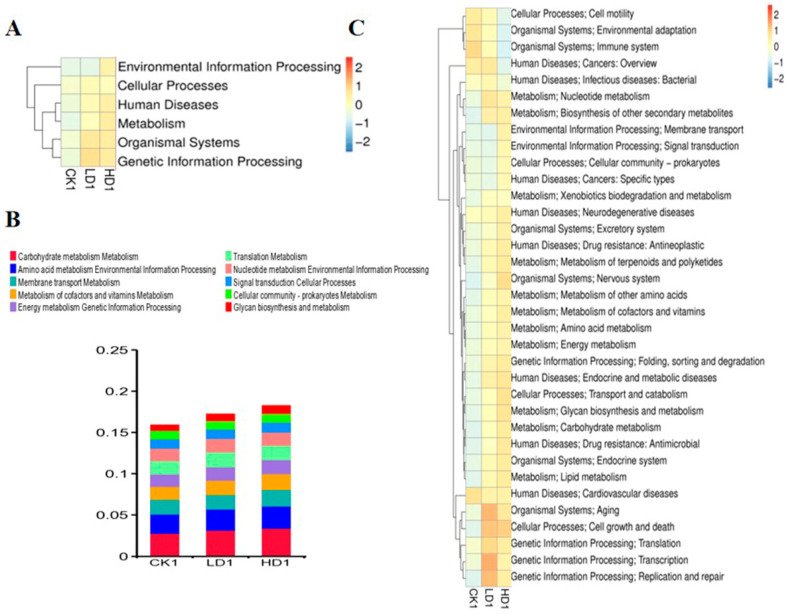
Heatmaps shows the effect of DON on mice gut microbiome function (KEGG database functional gene annotation at classification level 1 (**A**) and level 2 (**C**)). Relative abundance of mice gut bacteria functional genes at 2nd classification level in all treatment groups (**B**). CK1, LD1, and HD1 indicate the control, low-dose, and high-dose DON groups without recovery.

In addition to characterizing changes in the bacterial composition, the effects of DON exposure on the function of the mice cecum microbiome were defined. To this end, taxonomic assignment and functional annotations were carried out for the updated gene catalogue using 396,347 genes, using KEGG (v2018.01), eggNOG (v4.5), and CAZy (v2018.01) databases. For assessment at the functional level, 5238 KEGG orthologues and 19,373 eggNOG orthologue groups were identified in the updated gene catalogue. The KEGG database suggested the main activities of the annotated genes were associated with metabolism, genetic information processing, and environmental information at the first classification level. The results obtained reveal that the metabolism functional gene number increased with the low-dose DON exposure, but showed no increase in the high-dose group (Supplementary Table S9). The six functional gene clusters (environmental information processing, cellular process, human diseases, metabolism, organismal systems, genetic information processing) at the first classification level in the KEGG database showed changes to varying degrees (Figure 6A). DON exposure at low and high doses increased the expression of these six functional gene clusters to varying degrees. At the second classification level, the metabolism of co-factors and vitamin metabolism, translation metabolism, membrane transport metabolism, amino acid metabolism, environmental information processing, and carbohydrate metabolism were the top functional genes affected (Figure 6B). Other functional gene clusters show significant changes at the second classification level including genetic information processing (translation), cellular processes (transport and catabolism), human disease (neurodegenerative diseases), human diseases (infectious diseases (viral)), organismal systems (excretory system), and organismal systems (excretory system pathways (Figure 6C)). Two functional gene clusters, genetic information processing (replication and repair) and organismal systems (nervous system), at the second classification level, showed significant differences in both LD1 and HD1 groups compared with the CK1 group (Supplementary Table S10). At the KO level (KEGG ortholog group), we identified that k21572 (starch-binding outer membrane protein, SusD/RagB family), k03530 (DNA-binding protein HU-beta), k05349 (beta-glucosidase), k01190 (beta-galactosidase), k01992 (ABC-2 type transport system permease protein), k01206 (alpha-L-fucosidase), and k02025 (multiple sugar transport system permease protein) showed significant changes in abundance when comparing the CON group (CK1) and the Low-DON group (LD1) and/or the CON group (CK1) and the High-DON group (HD1) (Supplementary Figure S2A). These markers were mainly related to transport proteins, and this may indicate that DON exposure could affect gut microbiome transport protein synthesis. At the EC level (KEGG EC Number), 2.7.13.3, 1.97.1.4, 3.6.3.17, 2.7.7.6, 3.5.1.28 showed significant changes in the heatmap of EC numbers (Supplementary Figure S2B).

The functional characterization of the gut microbiome among different treatment groups was also validated by using eggNOG and CAZy databases. Chromatin structure and dynamics, intracellular trafficking, secretion, and vesicular transport and cell wall/membrane/envelope biogenesis show significant changes at the first classification pathway between the CON group (CK1) and both DON exposure groups (LD1 and HD1) based on the eggNOG database. Glycosyl transferases and glycoside hydrolases were the pathways mainly changed based on the CAZy database (Supplementary Figure S3A,B).

### 2.6. Prebiotic Inulin Administration May Impair the Function of the Mice Gut Microbiome after High-Level DON Exposure Compared to Spontaneous Recovery

**Figure 7:**
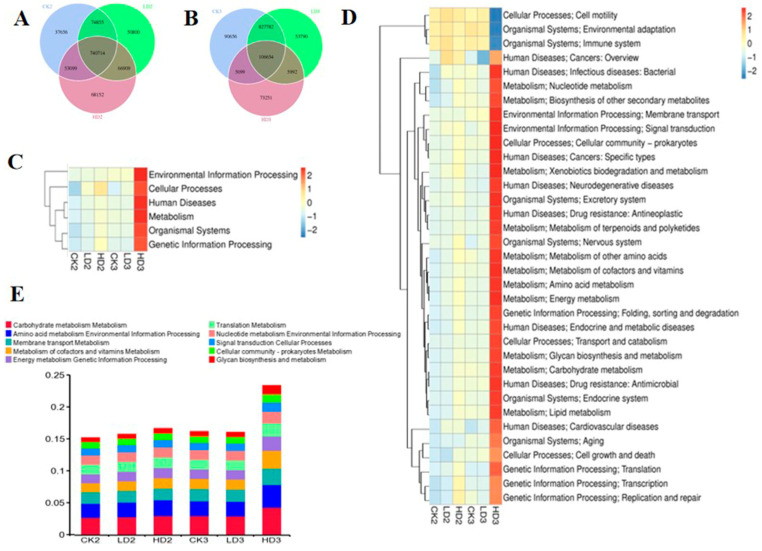
Venn flowers of gene numbers in CK2, LD2, and HD2 groups (**A**), and in CK3, LD3, and HD3 groups (**B**); Heatmap shows the effect of two ways of recovery after DON exposure on mice gut microbiome function (KEGG database functional gene annotation at classification level 1 (**C**) and level 2 (**D**,**E**)). CK2, LD2, HD2, and CK3, LD3, and HD3 indicate the control, low-dose, and high-dose DON groups with either spontaneous or inulin-supplemented recovery.

Microbial functional changes after DON exposure were also characterized after 2 weeks of spontaneous recovery (without intervention) or after 2 weeks of recovery with daily prebiotic inulin administration. After 2 weeks of spontaneous recovery or inulin intervention recovery, gene numbers in the Low-DON + Recover/Inulin groups (LD2 and LD3) and the High-DON + Recover/Inulin groups (HD2 and HD3) were changed compared to the respective control recover groups (CK2 and CK3, respectively). Two Venn flower figures showed common genes and unique genes in each group (Figure 7A,B). At the first classification level in the KEGG database, the relative abundance of metabolism gene clusters in the Low-DON + Recover group (LD2) still showed significant differences compared to the CON + Recover group (CK2) after two weeks of spontaneous recovery, whereas none of the above-mentioned six main functional gene clusters showed significant changes in the Low-DON + Inulin group (LD3) compared to the CON + Inulin group (CK3) after two weeks of inulin intervention recovery. None of the gene clusters in the High-DON + Recover group (HD2) showed significant differences compared to the CON + Recover group (CK2) after two weeks of spontaneous recovery, but in the HD3 group, the relative abundance of metabolism, genetic information processing, human diseases, and organismal system functional gene clusters showed significant differences compared to the corresponding control group (CK3) (Figure 7C and Supplementary Table S11). At the second classification level in the KEGG database, 11 functional gene clusters in the Low-DON + Recover group (LD2) showed significant differences compared to the CON + Recover group (CK2) after two weeks of spontaneous recovery, whereas none of the functional gene clusters in the Low-DON + Inulin group (LD3) showed significant differences compared to the CON + Inulin group (CK3). In the high-dose exposure groups, 8 of the functional gene clusters in the High-DON + Recover group (HD2) showed significant differences compared to the CON + Recover group (CK2) after two weeks of spontaneous recovery, and 31 of the functional gene clusters showed significant differences in the HD3 group compared to the CK3 group (Figure 7D and Supplementary Table S12). The top relative abundance of functional genes at the second classification level is shown in Figure 7E. Compared to the CON + Recover group (CK2), the metabolism of co-factors and vitamins, translation metabolism, membrane transport metabolism, amino acid metabolism, environmental information processing, and carbohydrate metabolism functional genes in the Low-DON + Recover group (LD2) and the CON + Recover group (CK2) were similar at the KEGG’s second classification level. Amino acid metabolism, environmental information processing, energy metabolism, and genetic information processing were still higher in the High-DON + Recover group (HD2) than in the CON + Recover group (CK2). On the KO level (Supplementary Figure S4A), K21572 (starch-binding outer membrane protein) and K12373 (hexosaminidase) increased, and K02003 (ATP-binding protein) decreased in group HD2 compared to groups CK2 and LD2, while most of other proteins in LD2 were identified to be present at levels similar to what was observed for the control group CK2. On the EC level (Supplementary Figure S4B), enzymes 1.6.5.3 and 3.6.4.12 were significantly decreased, but 2.1.1.72 significantly increased in group HD2 compared to the control recovery group (CK2). The functional characterization of the gut microbiome validated by using eggNOG (Supplementary Figure S5A) and CAZy (Supplementary Figure S5B) databases revealed that extracellular structures’ pathway genes were still increased in group LD2 compared to the control group (CK2). Intracellular trafficking, secretion, and vesicular transport were increased in group HD2 compared to CK2 at the classification level 1 of eggNOG and CAZy databases. Transcriptional regulator decreased in group LD2, and ABC transporter (permease) increased in group HD2 compared to CK2. The CAZy database showed that the pathways in group LD2 were similar to those in the CON + Recover group (CK2) after 2 weeks of natural recovery, except for the glycoside hydrolases in group HD2 that were still increased compared to the CON + Recover group (CK2).

In brief, inulin supplementation was helpful in the recovery of mice gut microorganism functions after low DON exposure, but the results were opposite in the high-dose group where differences appeared to become larger upon inulin recovery.

## 3. Discussion

We used whole genome gene sequencing to study the impact of DON exposure on the gut microbiome. The data obtained clearly show that DON exposure induced an obvious change in the gut microbiome composition of mice. Our observation and taxonomic annotation of the gut microbiota in mice were not fully consistent with a previous report by Wang et al. [19]. Wang et al. reported viruses as being the most dominant phylum across all samples in the control group, Firmicutes being the most abundant in the low-dose group (2.0 mg/kg body weight of DON), and Bacteroidetes, Firmicutes, and Deferribacteres being the most abundant in the high-dose group (5.0 mg/kg body weight of DON) in a mouse model. In our study, Firmicutes, Bacteroidetes, and Verrucomicrobia were predominant in the gut bacteria of mice at the phylum level, followed by Proteobacteria, Deferribacteres, and Actinobacteria. Only 0.1% of the detected complete genes were annotated as coming from viruses, pointing at a substantial difference compared to the study from Wang et al. [19]. The high prevalence of Firmicutes and Bacteroidetes was consistent between the two studies. A possible assumed reason for the discrepancies between the two metagenomic studies might be related to the fact that the mice used in the Wang et al. study were virus-infected, leading to different outcomes in metagenome composition. In addition to this, feed, water, and living environment exposure of the mice during the study may also cause differences in gut microbiome composition.

In this study, we observed that the relative abundance of Verrucomicrobia increased with DON exposure in a dose-dependent way. Verrucomicrobia is a phylum of Gram-negative bacteria, that is occasionally observed in humans. Broad-spectrum antibiotic therapy can cause high-level colonization of the human gut by Verrucomicrobia [20]. However, the mechanism underlying this colonization by Verrucomicrobia was not clear. A human progeria patient exhibited a similar tendency in Verrucomicrobia abundance, and long-lived humans such as centenarians were shown to display a substantial increase in Verrucomicrobia and a reduction in Proteobacteria [21].

DON exposure increased the relative abundance of species *A. muciniphila*, *L.bacterium* 28-4, and *H. hathewayi. A. muciniphila* belongs to the phylum Verrucomicrobia, and is a strict anaerobic, Gram-negative species of bacteria. *A. muciniphila* is one of the most abundant single species in intestinal microbiota, and is considered as a promising next-generation beneficial microbe [22]. It is a highly specialized bacteria because of its ability to use mucins as a sole source of carbon and nitrogen [23]. Meanwhile, *A. muciniphila* can stimulate mucin expression and mucus secretion by positive feedback [23]. This property reveals a competitive advantage under conditions of nutrient deprivation, such as, for example, fasting, malnutrition, or total parenteral nutrition. *A. muciniphila* releases easily available short-chain fatty acids, acetate, and propionate for the host as a result of mucin degradation. *A. muciniphila* was inversely associated with obesity, diabetes, cardiometabolic diseases, and low-grade inflammation [22]. A previous study observed that mice treated with *A. muciniphila* gained less weight, improved glucose tolerance, and exhibited insulin resistance under hyperlipidic diet conditions [24]. The administration of specific dietary components or pharmaceutical treatments affected the level of *A. muciniphila,* such as polyphenols, fructo-oligosaccharide, conjugated linoleic acid, oat bran, and metformin intake [25]. Our study shows that DON exposure can also increase the abundance of *A. muciniphila.* The reason may be due to the fact that DON causes damages to the intestinal barrier function, which may simulate *A. muciniphila* growth to repair the damages. The mode of action underlying this observation is of interest for future exploration.

*L. bacterium* 28-4 was enriched upon DON exposure, which was previously shown to be enriched in pigs with low residual feed intake (high feed efficiency) [26]. *L. bacterium* 28-4 was also investigated to be the dominant species in mice with resistance to high-fat diet-induced obesity [27]. A study conducted in pathogen-free C57BL/6J mice showed that pomegranate fruit polyphenols enriched *A. muciniphila*, *L. bacterium* 28-4, and three other species. These enriched species were negatively correlated with body weight, glucose, triglycerides, and total cholesterol levels in serum [28].

*H. hathewayi* was also enriched upon DON exposure, belonging to *Lachnospiraceae*, which was less likely to cause human diseases, and was proven to be a common fecal flora commensal [29]. *H. hathewayi* was reported to be involved in the progress of some diseases, such as unruptured intracranial aneurysm [30]. The abundance of *H. hathewayi* was positively associated with taurine concentration in mice and human circulation, and oral gavage with it can normalize taurine levels in serum. The taurine could protect mice from the formation and rupture of intracranial aneurysms, and *H. hathewayi* was negatively associated with the pathogenesis of the disease [30].

We noticed that the relative abundance of the species *Oscillibacter* sp. 1-3 decreased with DON exposure, but increased in the recovery groups (LD2 and HD2) compared to the levels of the control group (CK2) after 14 days of natural recovery in this study. *Oscillibacter* was proven to be causally linked to decreased triglyceride in the blood (Liu et al., 2022) [31]. *Oscillibacter* was also associated with obesity; the relative abundance of *Oscillibacter* was reduced in obese individuals [32]. *Oscillibacter* spp. and *Akkermansia* spp. showed a significantly negative correlation with lipopolysaccharide (LPS) levels in plasma [33]. Speculatively, the decreased abundance of *Oscillibacter* sp. 1-3 over time upon DON exposure could give rise to high levels of lipopolysaccharide (LPS) concentration in plasma. *Oscillibacter* belongs to butyrate-producing bacterial families, and was decreased in patients with early hepatocellular carcinoma (HCC) [34]. The genome of *Oscillibacter* sp. 1-3 encodes tryptophanase, capable of synthesizing indole from tryptophan. *Oscillibacter* sp. 1-3 and *Firmicutes bacterium* ASF500 have important characteristics in enhancing intestinal epithelial barrier functions and immunity [35]. Accordingly, the restructuring of microbial populations responding to DON exposure is likely the cause of perturbation of the intestinal barrier function. In addition, *Oscillibacter* was considered a harmful bacteria [36]. It was reported that *Oscillibacter* was associated with trimethylamine oxide (TMAO), which is considered as a risk factor for cardiovascular and cerebrovascular disease [37].

Gut microbiota resilience and natural recovery were reported after antibiotic administration [38]. Natural recovery of gut microbiota changes after mycotoxin exposure has not been investigated before. In this research, we showed that upon 2 weeks of natural recovery after DON exposure, mice gut microbiota communities returned to their initial state and fully recovered, especially in the low-dose exposure group. The Low-DON + Recover group (LD2) recovered better than the High-DON + Recover group (HD2) based on both the composition and function of the gut microbiome. In contrast to the low-dose DON exposure group, the changes observed upon high-dose DON exposure did not fully return to control levels in the 2-week spontaneous recovery period.

Dynamics of the microbial community are strictly correlated with disease development by altering the metabolic processes and/or the immune responses of the host [39]. More and more prebiotics have been characterized to enhance the beneficiary effects of gut microbiota substantially. One of the most commonly applied prebiotics in the restoration of intestinal microbiota is inulin. Inulin is extracted from chicory root, and is normally commercially used to stimulate the growth of *Bifidobacterium* and *Lactobacillus* [40]. In an earlier study conducted by Lin et al. [41], inulin showed the greatest elimination ability on a single-course amoxicillin-induced disruption of mice gut microbiota abundance and diversity enrichment. In our study, we observed a positive effect of inulin on reviving the destructed DON-induced gut microbiota disruption for the low-dose exposure group. However, in the high-dose group, the inulin supplementation showed a negative effect on gut microbiome recovery. It even further decreased the diversity of the mice gut microbiome, and led to dysbiosis compared with spontaneous recovery. After high-dose DON exposure, the mice gut microbiome composition and function were changed, and the supplementation of inulin appeared to amplify the differences in the gut microbiome by an as-yet unknown mode of action. Additionally, immediately following the treatment, the phylum Verrucomicrobia was dose-dependently increased in the DON groups (LD1 and HD1) compared to the corresponding control (CK1) at the cost of the Fermicutes. Furthermore, upon 14 days of natural recovery, this increase in Verrucomicrobia was not fully annihilated in the High-DON + Recover group (HD2) compared to the corresponding control (CK2).

While main research efforts to elucidate the impact of DON treatment on gut microbiota have mainly focused on community shifts [17], little is known about the functional consequences of these shifts for the cross-talk between gut microbial metabolism and host responses. In this study, we characterized the genes’ functional annotation in all experimental groups. All of the six functional gene clusters (environmental information processing, cellular process, human diseases, metabolism, organismal systems, genetic information processing) involved in the response to DON exposure exhibited differently in terms of their abundance. It is apparent to see that natural recovery can repair the gene function loss under low-dose DON exposure after 2 weeks. Unexpectedly, inulin supplements showed no obvious beneficial effects on the recovery of gene functions after DON perturbation.

## 4. Conclusions

In summary, our research data highlight the profound influence of 2 weeks of repeated DON exposure on the mice gut microbiota and functional genes. DON exposure enhanced the relative abundance of *A. muciniphila*, *B. vulgatus*, *H. hathewayi,* and *L. bacterium* 28-4, while relative abundances of *M. schaedleri*, *Pseudoflavonifractor* sp. An85, *F. prausnitzii*, *F. bacterium* ASF500, *F. plautii*, *Oscillibacter* sp. 1-3, and *uncultured Flavonifractor* sp. were decreased. The specific microbial taxonomic profile and functional genes were comparable between control and the low-dose DON groups after 2 weeks of natural recovery, indicating that most of the gut microbiome species in mice regrow to initial levels after low-dose DON exposure during the 2-week recovery. In both the low- and high-dose DON group, about 10 percent of the changed gut microbiome after exposure still existed after 2 weeks of natural recovery. Inulin administration appeared to promote the recovery of the gut microbiome and functional genes after low-dose DON exposure, but not after high doses, for which changes were exacerbated by inulin-supplemented recovery. Overall, this study may help to understand the microbiome shifts after mycotoxin exposure, and to choose the appropriate way to help the reconstruction of the gut microbiome upon changes induced by dietary contaminants.

## 5. Materials and Methods

### 5.1. Chemicals and Solutions

DON was purchased from Beijing Meizheng Testing Co., Ltd. (Beijing, China), purity ≥ 98%. Inulin was purchased from Shanghai Sangon Company (Shanghai, China). Mice daily feed was prepared by Xiaoshuyoutai Co., Ltd. (Beijing, China), according to AIN-93M standard.

### 5.2. Animals

A number of 72 BALB/c female mice, aged 6–7 weeks, weight 20–22 g (Vital River, Beijing, China), were housed at room temperature (25 ± 2) °C, under 12 h light and dark cycles. The mice were allowed access to food and water ad libitum, and were maintained with 4 animals per cage. The mice were housed in a standard SPF facility of the Institute of Food Science and Technology (IFST), Chinese Academy of Agricultural Sciences (CAAS). All the animal experiments were carried out under the approval and supervision of the ethics committee of IFST, CAAS (No. JGS-20181005). All the animal experiments were in accordance with the NIH Guide for the Care and Use of Laboratory Animals.

### 5.3. Animal Groups and Treatments

After 1 week of acclimation, 72 female mice were assigned to 9 different treatments randomly, 8 animals per group. 200 μL purified water with or without 2 or 5 mg/kg bw/day DON was administered to animals via intragastric infusion (IG) once daily. Chemical DON was suspended in purified water using ultrasound for 15 min.

To study the effects of DON on mice gut microbiota, the treatment groups were as follows: (1) CON group (CK1): purified water for 14 days; (2) Low-DON group (LD1): 2 mg/kg bw/day DON for 14 days; (3) High-DON group (HD1): 5 mg/kg bw/day DON for 14 days.

To study the 2-week recovery period after DON exposure, additional groups were treated as follows: (4) CON + Recover group (CK2): purified water for 14 days, followed by natural recovery for 14 days; (5) Low-DON + Recover group (LD2): 2 mg/kg bw/day DON for 14 days, followed by purified water and regular diet for 14 days; (6) High-DON + Recover group (HD2): 5 mg/kg bw/day DON for 14 days, followed by purified water and regular diet for 14 days. (7) CON + Inulin group (CK3): purified water for 14 days, followed by purified water and an inulin diet (5% inulin addition to AIN-93M) for 14 days; (8) Low-DON + Inulin group (LD3): 2 mg/kg bw/day DON for 14 days, followed by purified water and an inulin diet for 14 days; (9) High-DON + Inulin group (HD3): 5 mg/kg bw/day DON for 14 days, followed by purified water and an inulin diet for 14 days.

Mice body weight was measured every four days over the whole duration of the study. The mice were sacrificed after anesthesia on day 15 (groups 1–3) or day 29 (groups 4–9). Whole blood was collected from the mice orbit after sacrifice. Plasma was collected by centrifugation (3000 rpm, 20 min, 4 °C), and stored at −80 °C. Intestinal content (cecum) was collected after sacrifice, and stored at −80 °C until further analysis.

### 5.4. DNA Extraction, Library Construction and Sequencing

Frozen cecum contents from each group were used for metagenomics study. Genomic DNA was extracted with the QIAamp DNA stool mini kit (Qiagen, Valencia, CA, USA) following the protocol provided by the supplier. Extracted genomic DNA (2 ng/μL) was used for library preparation. The purity and integrity of the DNA was determined with a nanodrop (ND-1000) spectrophotometer (Nanodrop Technologies, Wilmington, DE, USA) through 1% agarose gel electrophoresis (AGE). DNA concentration was measured using a Qubit^®^ dsDNA Assay Kit in a Qubit^®^ 2.0 Fluorometer (Life Technologies, Carlsbad, CA, USA). Samples with A260/A280 values between 1.8–2.0 and a total mass of DNA above 1 μg were collected for metagenomic sequencing and used to construct the library. Sequencing libraries were generated using a NEBNext^®^Ultra™ DNA library Prep Kit for Illumina (NEB, lpswich, MA, USA) following the manufacturer’s recommendations and index codes were added to attribute sequences to each sample. Briefly, the DNA sample was sonicated into fragments of 350 bp on average, then DNA fragments were end-polished, A-tailed, and ligated with the full-length adaptor for Illumina sequencing with further PCR application. Finally, PCR products were purified (AMPure XP system) and libraries were analyzed for size distribution with an Agilent 2100 Bioanalyzer and quantified using real-time PCR. The clustering of the index-coded samples was performed on a cBot Cluster Generation System according to the manufacturer’s instructions. After cluster generation, the library preparations were sequenced on an Illumina HiSeq platform and paired-end reads were generated.

### 5.5. Sequencing Data Pretreatment and Metagenome Assembly

The raw data obtained from the Illumina HiSeq sequencing platform using Readfq were processed to acquire the clean data for subsequent analysis. Considering that the possibility of host pollution of the samples may exist, clean data were blasted to the host database which, by default, uses Bowtie 2.2.4 software to filter out the reads that are of host origin. The clean data were assembled and analyzed by SOAP de novo software (V2.04).

### 5.6. Gene Prediction and Abundance Analysis

The Scaftigs (≥500 bp) assembled from both single and mixed assemblies were all predicted by the ORF by MetaGeneMark (V 2.10) software, and the length information for fragments shorter than 100 nt was filtered from the predicted result with default parameters. CD-HIT software (V4.5.8) was adopted for redundancy and to obtain a unique initial gene catalogue. The clean data of each sample were mapped to an initial gene catalogue using Bowtie 2.2.4. The basic information statistics, core-pan gene analysis, correlation analysis of samples, and Venn figure analysis of the number of genes were all based on the abundance of each gene in the respective sample.

### 5.7. Taxonomic Assignment of Genes

DIAMOND software (V0.9.9) was used to analyze the unigenes via BLAST against the sequences of bacteria, fungi, archaea, and viruses which were all extracted from the NR database (Version: 20180102, https://www.ncbi.nlm.nih.gov/, accessed on 10 February 2019). For each sequence’s blast result, the best Blast Hit was used for subsequent analysis. A functional database including the KEGG database, eggNOG database, and CAZy database was used in this study. The least common ancestors (LCA) algorithm was applied to the system classification using MEGAN software to characterize the species annotation information of sequences. PCA (R ade4 package, Version 2.15.3) and NMDS (R vegan package, Version: 2.15.3) decrease dimension analyses were based on the abundance table of each taxonomic hierarchy.

### 5.8. Statistical Analysis

Results were expressed as mean ± SEM. Significances of differences between two or multiple groups were determined using a two-sided unpaired Student’s *t*-test or one-way analysis of variance (ANOVA). All analyses were performed at least in triplicate. Statistical analyses were performed using GraphPad Prism v9.0. *p* < 0.05 was considered to be statistically significant. Metastats and LEfSe analysis were used in Metastats analysis for each taxonomy and to obtain the *p* value, then the Benjamini and Hochberg false discovery rate procedure was used to correct the *p* value and acquire the *q* value. LefSe analysis was conducted by using LEfSe software. Random forest (RandoForest) (P pROC and randomForest packages, Version 2.15.3) was used to construct a random forest model. Important species were screened out by MeanDecreaseAccuracy and MeanDecreaseGin, and the receiver operating characteristic curve was plotted for cross-analysis validation of each model. The heat maps were generated using R language to visualize the gut microbiome differences between treatment groups.

## Data Availability

The data that support the findings of this study are available from the corresponding author upon reasonable request.

## Supplementary Materials

The following supporting information can be downloaded at: https://www.mdpi.com/article/10.3390/toxins15040243/s1, Figure S1: A–E. The identified top 20 gut bacteria assigned at class, order, family, genus, and species levels. CKn, LDn and HDn indicate the control, low dose and high dose DON groups of the respective series; Figure S2. KEGG database functional gene annotation (KO (A), and ec (B). CKn, LDn and HDn indicate the control, low dose and high dose DON groups without recovery.; Figure S3. Heatmap of egg NOG database pathways (level 1) (A); Heatmap of CAZy database pathways (level 1) (B). CKn, LDn and HDn indicate the control, low dose and high dose DON groups without recovery.; Figure S4. KEGG database functional gene annotation (KO (A), and ec (B). CKn, LDn and HDn indicate the control, low dose and high dose DON groups with either spontaneous or inulin supplemented.; Figure S5. Heatmap of egg NOG database pathways (level 1) (A); Heatmap of CAZy database pathways (level 1) (B). CKn, LDn and HDn indicate the control, low dose and high dose DON groups with either spontaneous or inulin supplemented.
