# Supplementary material for: Metagenome Analysis Identifies Microbial Shifts upon Deoxynivalenol Exposure and Post-Exposure Recovery in the Mouse Gut
Source: Toxins (Basel). 2023 Mar 25;15(4):243. doi: 10.3390/toxins15040243 (PMC10142982; doi:10.3390/toxins15040243)
Supplement: Supplementary file 1 [file toxins-15-00243-s001.zip › toxins-2270418-supplementary.pdf]

Supplementary Materials

Supplementary Fig. 1

A

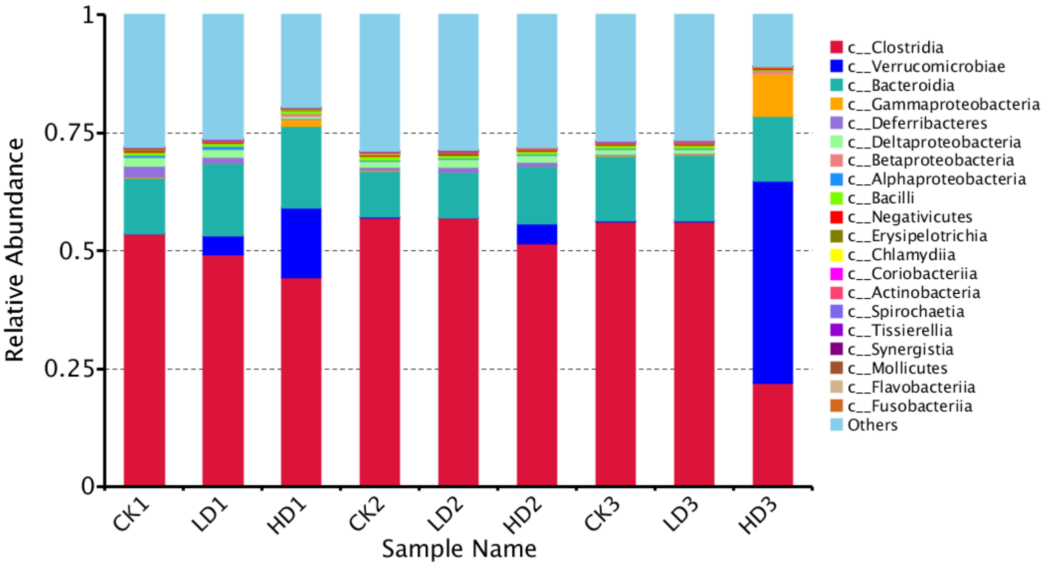

B

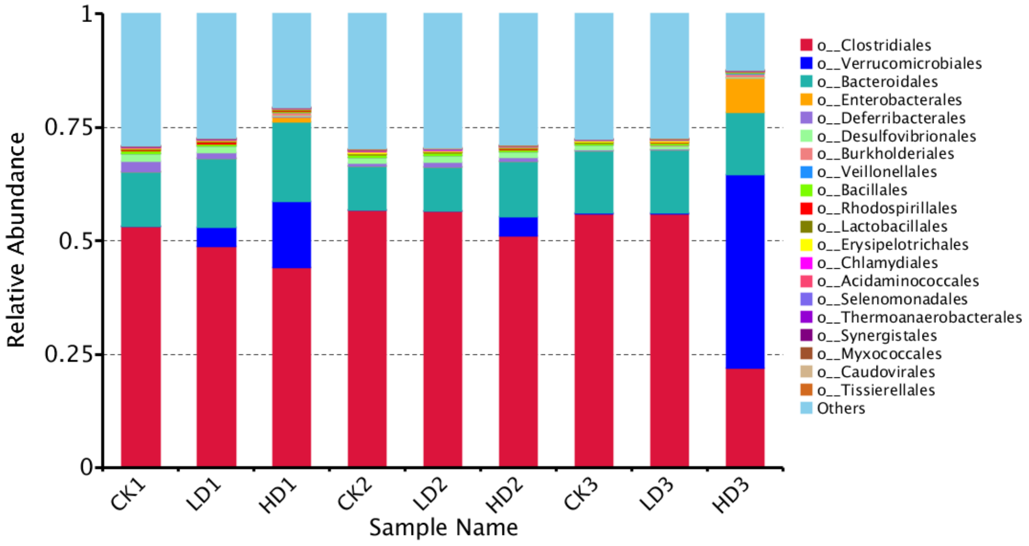

C

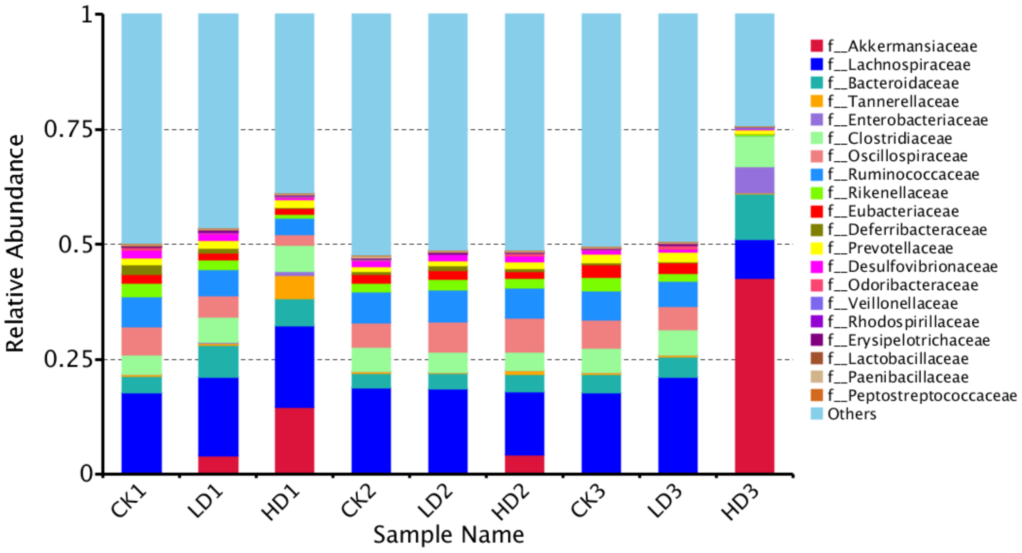
Relative Abundance
0
0.25
0.5
0.75
1
CK1
LD1
HD1
CK2
LD2
HD2
CK3
LD3
HD3
Sample Name
f__Akkermansiaceae
f__Lachnospiraceae
f__Bacteroidaceae
f__Tannerellaceae
f__Enterobacteriaceae
f__Clostridiaceae
f__Oscillospiraceae
f__Ruminococcaceae
f__Rikenellaceae
f__Eubacteriaceae
f__Deferribacteraceae
f__Prevotellaceae
f__Desulfovibrionaceae
f__Odoribacteraceae
f__Veillonellaceae
f__Rhodospirillaceae
f__Erysipelotrichaceae
f__Lactobacillaceae
f__Paenibacillaceae
f__Peptostreptococcaceae
Others

D

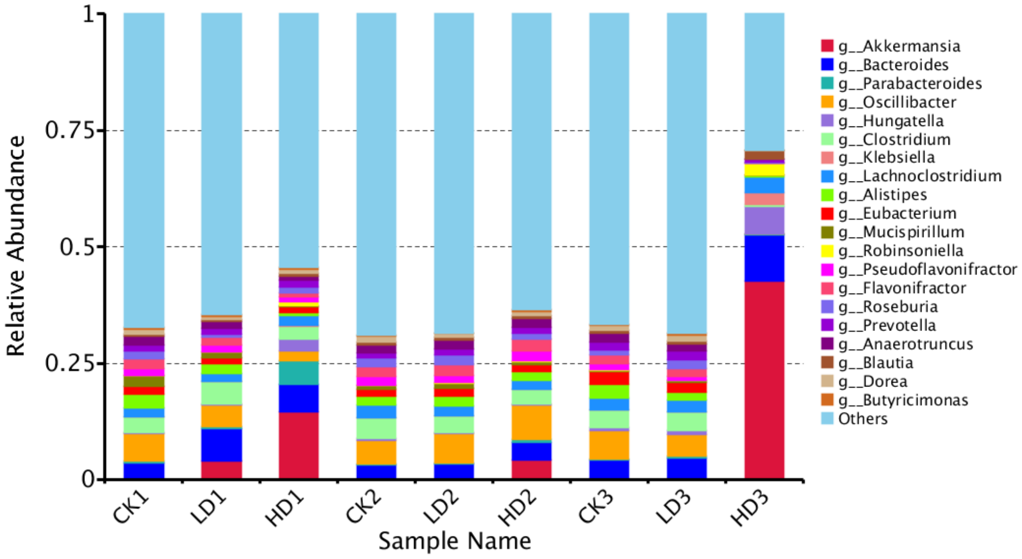
Relative Abundance
0
0.25
0.5
0.75
1
CK1
LD1
HD1
CK2
LD2
HD2
CK3
LD3
HD3
Sample Name
g__Akkermansia
g__Bacteroides
g__Parabacteroides
g__Oscillibacter
g__Hungatella
g__Clostridium
g__Klebsiella
g__Lachnoclostridium
g__Alistipes
g__Eubacterium
g__Mucispirillum
g__Robinsoniella
g__Pseudoflavonifractor
g__Flavonifractor
g__Roseburia
g__Prevotella
g__Anaerotruncus
g__Blautia
g__Dorea
g__Butyricimonas
Others

E

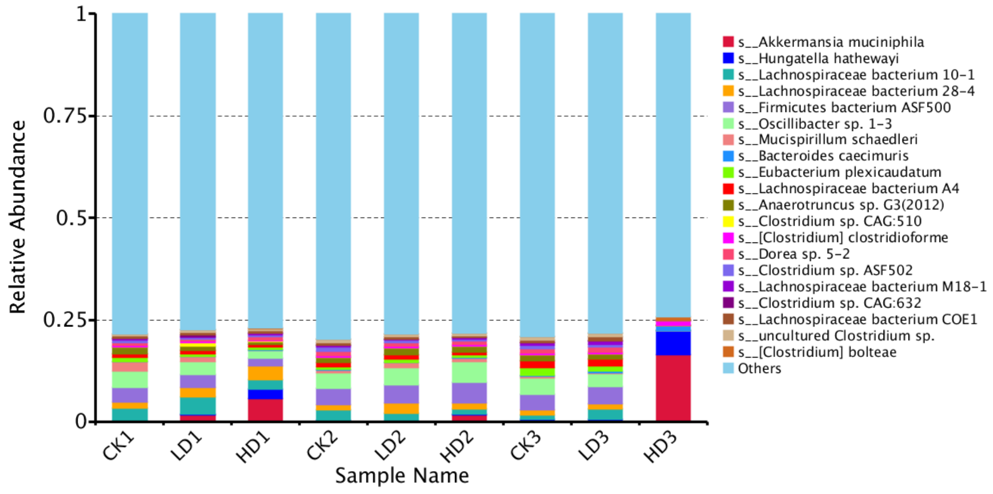

Figure S1. A–E. The identified top 20 gut bacteria assigned at class, order, family, genus, and species levels. CKn, LDn and HDn indicate the control, low dose and high dose DON groups of the respective series. .

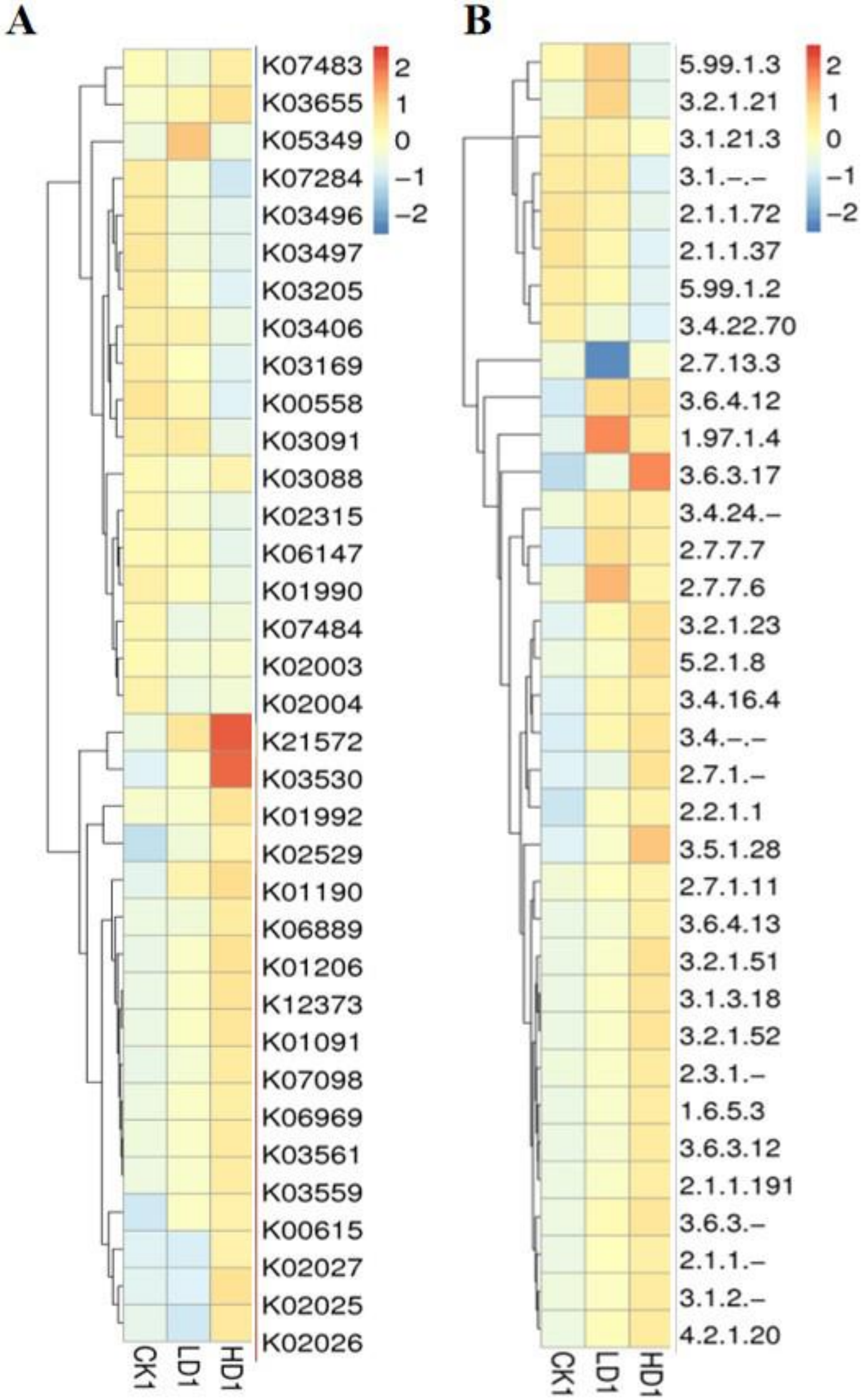

Figure S2. KEGG database functional gene annotation (KO (**A**), and ec (**B**). CKn, LDn and HDn indicate the control, low dose and high dose DON groups without recovery.

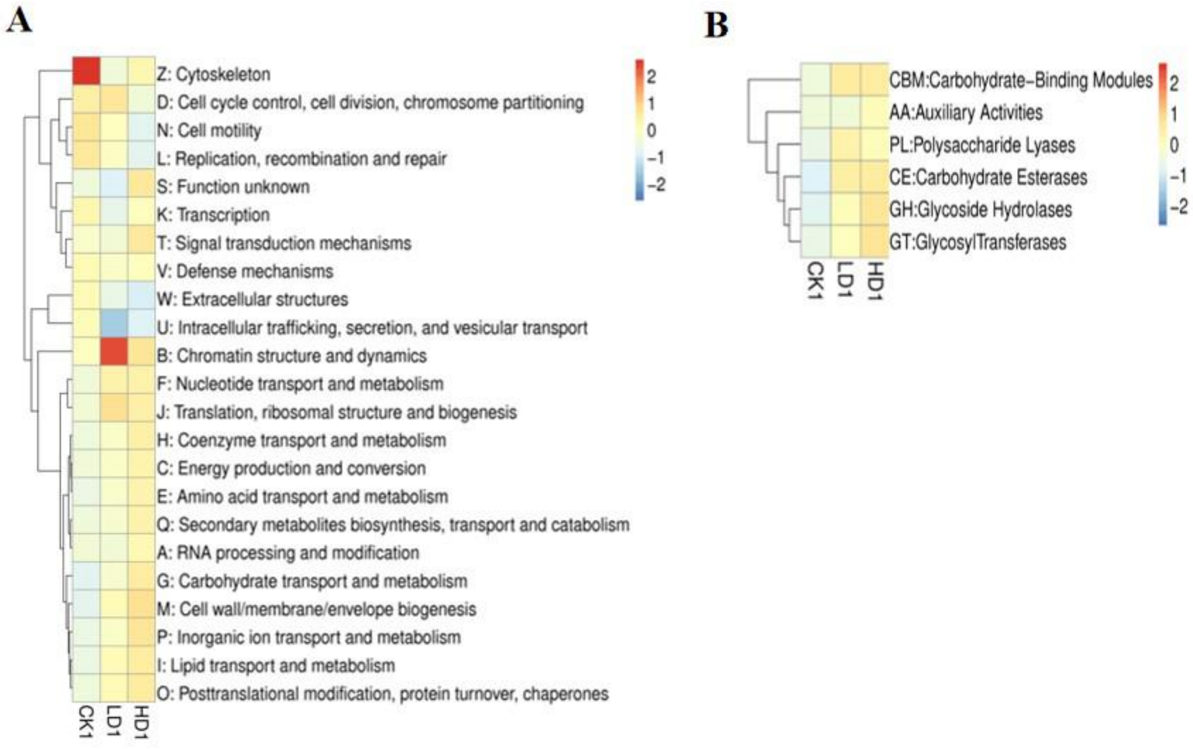

Figure S3. Heatmap of egg NOG database pathways (level 1) (**A**); Heatmap of CAZy database pathways (level 1) (**B**). CKn, LDn and HDn indicate the control, low dose and high dose DON groups without recovery.

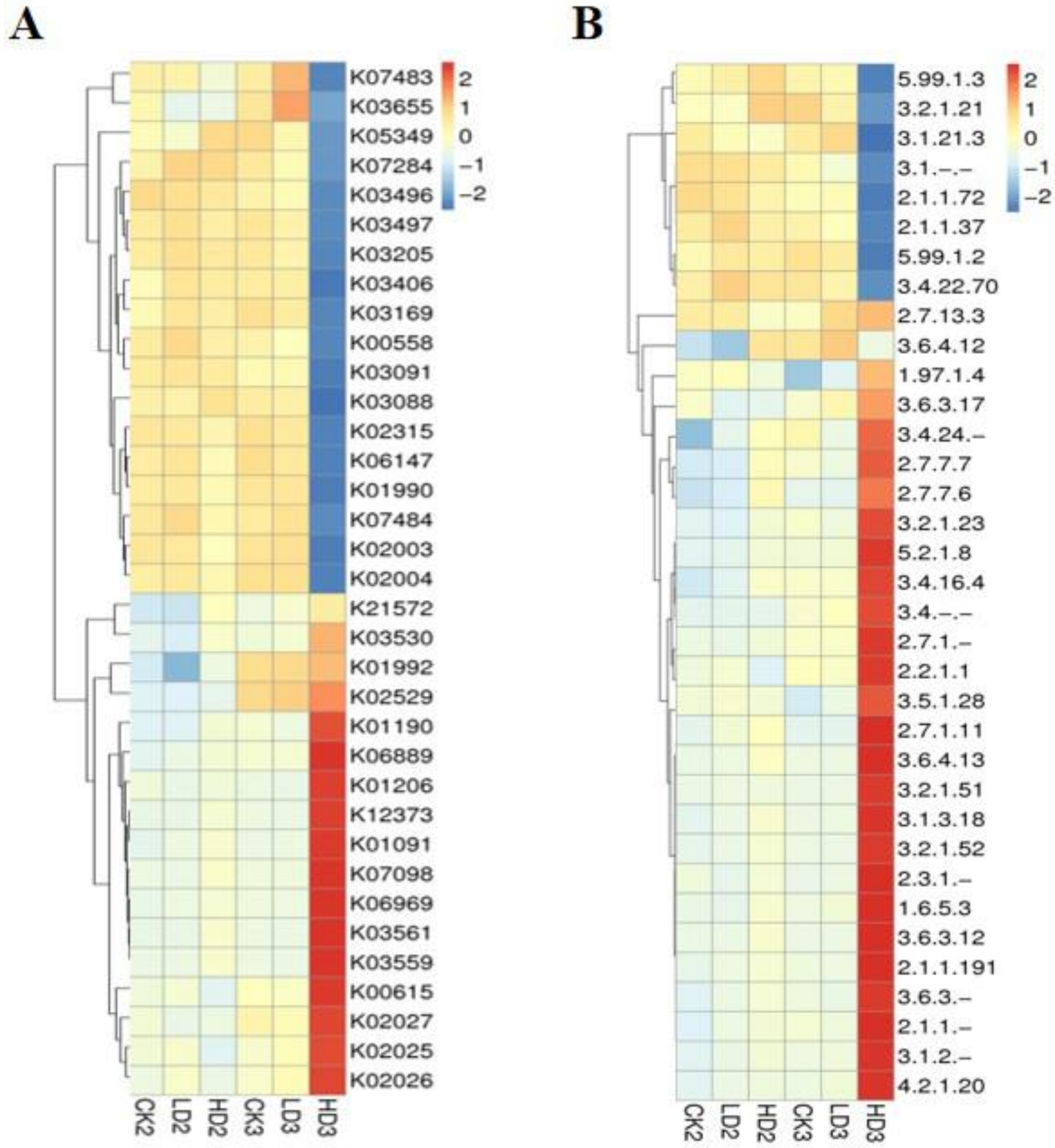

Figure S4. KEGG database functional gene annotation (KO (**A**), and ec (**B**). CKn, LDn and HDn indicate the control, low dose and high dose DON groups with either spontaneous or inulin supplemented.

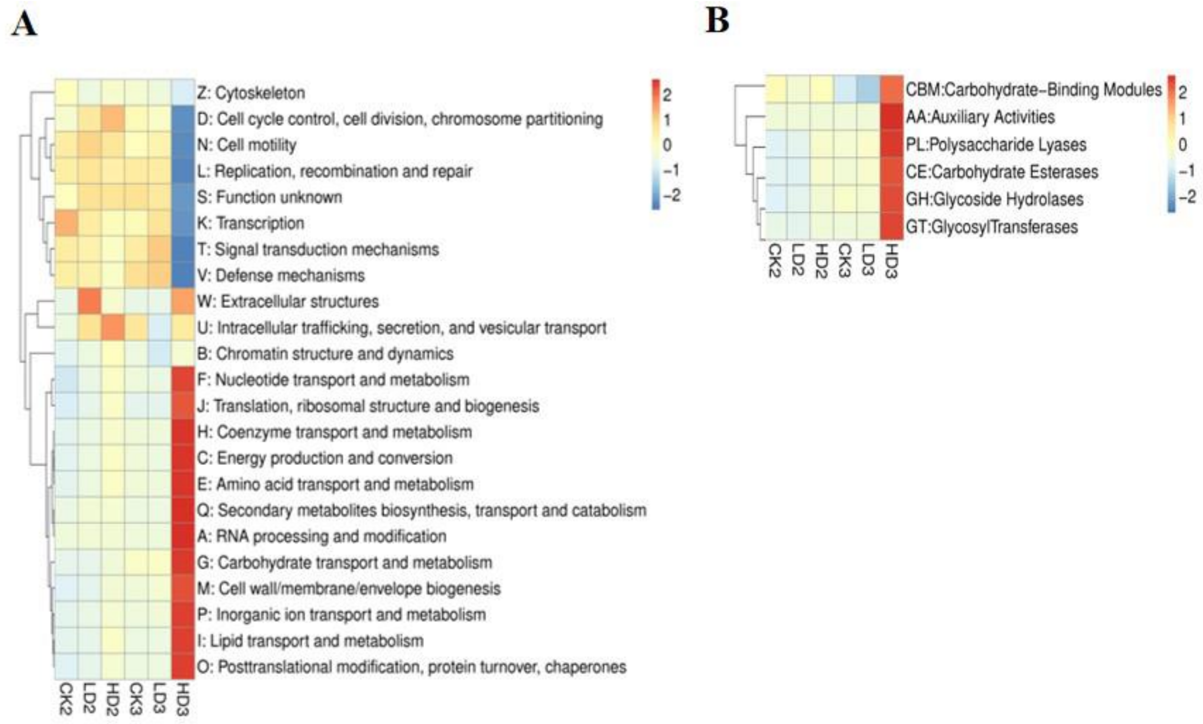

Figure S5. Heatmap of egg NOG database pathways (level 1) (**A**); Heatmap of CAZy database pathways (level 1) (**B**). CKn, LDn and HDn indicate the control, low dose and high dose DON groups with either spontaneous or inulin supplemented.
